# Adversity, emotion recognition, and empathic concern in high-risk youth

**DOI:** 10.1371/journal.pone.0181606

**Published:** 2017-07-24

**Authors:** Jodi A. Quas, Kelli L. Dickerson, Richard Matthew, Connor Harron, Catherine M. Quas

**Affiliations:** 1 Department of Psychology and Social Behavior, University of California Irvine, Irvine, California, United States of America; 2 Department of Planning, Policy, and Design, University of California Irvine, Irvine, California, United States of America; 3 Department of Social Ecology, University of California Irvine, Irvine, California, United States of America; 4 Bluefish Dental, Bend Oregon, United States of America; Universite de Bretagne Occidentale, FRANCE

## Abstract

Little is known about how emotion recognition and empathy jointly operate in youth growing up in contexts defined by persistent adversity. We investigated whether adversity exposure in two groups of youth was associated with reduced empathy and whether deficits in emotion recognition mediated this association. Foster, rural poor, and comparison youth from Swaziland, Africa identified emotional expressions and rated their empathic concern for characters depicted in images showing positive, ambiguous, and negative scenes. Rural and foster youth perceived greater anger and happiness in the main characters in ambiguous and negative images than did comparison youth. Rural children also perceived less sadness. Youth’s perceptions of sadness in the negative and ambiguous expressions mediated the relation between adversity and empathic concern, but only for the rural youth, who perceived less sadness, which then predicted less empathy. Findings provide new insight into processes that underlie empathic tendencies in adversity-exposed youth and highlight potential directions for interventions to increase empathy.

## Introduction

In recent years, scientific research, policy, and even public attention has turned toward attempting to understand how some of the most fundamental social processes that make us human—compassion, empathy, and concern for others—operate in a world filled with vast poverty, desperation, and violence. These processes are core to our ability to connect with one another, form close relationships, and engage with others; and are believed to underlie a range of prosocial and altruistic tendencies [1, 2]. Despite recognition of the critical role that empathy and related processes play in human lives, questions remain about precisely how empathy functions in contexts defined by extreme adversity and challenge, particularly in childhood, a time when emotional functioning generally, including possibly empathy, is undergoing rapid change.

In the current investigation, we examined empathic concern in high-risk children and adolescents growing up in a small, impoverished country in the southern part of Africa, Swaziland. Our primary questions were first whether exposure to chronic adversity was associated with reduced empathic concern, and second, whether the association between adversity and empathic concern varied as a function of the youth’s ability to recognize others’ emotions.

Swaziland was an ideal data collection site for several reasons. The country, like others in the region, is highly impoverished, with a vast majority of the population living in conditions of extreme poverty. Swaziland also has one of the highest rates of HIV/AIDs in the world [3], which, when combined with commonly co-occurring diseases (e.g., tuberculosis), contributes to very high rates of illnesses and deaths in the population. For instance, the country’s infant and child mortality rates are among the highest in the world, and the average lifespan, under 50, is among the lowest [4]. Thus, large numbers of children are growing up with ill or deceased parents, siblings, and other family members, experiences that are accompanied by uncertainty, inconsistent caregiving, and challenge [5]. Finally, the ethnicity and religion of the population are largely homogenous (97% Black; 97% Catholic or Zionist), and the country has not endured any major sociopolitical, ethnic, or religious conflict for several generations. Thus, Swazi children have been exposed to high levels of chronic adversity as reflected in poverty and family stress, but not unpredictable violence, which may affect emotional processes in ways that are different from chronic but somewhat predictable adversity [6].

Empathy generally refers to one’s tendency to share or respond to others’ emotions or feelings [7, 8]. An implicit assumption in this definition, and an assumption that has yet to be adequately tested, is that empathic individuals easily and consistently recognize the emotions being displayed or felt by others [9–11]. On the one hand, perhaps testing this assumption is unnecessary: basic emotion recognition emerges very early in development, and even relatively young children can accurately label and respond to a range of emotional displays in others [12, 13]. On the other hand, however, experiential and developmental factors play a role in emotion recognition tendencies [14], particularly in childhood. Insofar as variations exist in how well children recognize emotions being displayed by others, such variations may affect whether or not children seem empathic in turn.

In particular, in a largely separate literature, research has focused on how exposure to compromised home environments, such as those defined by neglect or abuse by parents, severe deprivation, or parental mental illness, affects children’s interpretations and responses to others’ displays of negative emotions, most especially anger and sadness. With regard to anger, findings have been fairly consistent in revealing heightened sensitivity to anger among adversity-exposed children. For instance, children who have been physically abused often recognize anger more quickly and accurately than children without a history of physical abuse [15, 16]. At the same time, this sensitivity seems to extend to situations in which negative emotions are perhaps less clear, with physically abused children tending to “recognize” or see anger in emotionally ambiguous expressions and situations [17–20]. Research with children raised in institutionalized foster care settings has revealed similarly liberal tendencies toward perceiving anger [21], suggesting that chronic exposure to neglectful or inconsistent caregiving and violence may all contribute to anger bias tendencies in children.

Findings concerning adversity-exposed children’s perceptions of other emotions, particularly sadness, are less consistent than findings concerning anger, but, when findings do emerge, they tend to reveal deficits or difficulties in emotion recognition tendencies among such children, as well [18, 21, 22]. In one investigation, for example, Wismer Fries and Pollak [21] compared children who had been raised in Eastern European institutions and children who had always lived with their biological parents. Although the institutionalized children had been subsequently adopted, they nonetheless were less accurate than the comparison children when attempting to identify happy, sad, and fearful expressions in photographs. In an earlier investigation, Pollak et al. [18] found similar results: compared to non-maltreated children, neglected children had difficulty discriminating among emotional expressions, and physically abused children were poorer at recognizing sadness. Finally, even youth exposed to civil war, including former child soldiers, appear to show reduced accuracy in identifying sadness in facial expressions, and in one investigation, child soldiers tended to mislabel sadness in others as anger [23].

Theoretically, when caregivers are inconsistent or unavailable, children lack sufficient input to learn to recognize emotions broadly. They instead develop a heightened sensitivity to emotions that are most critical for their daily lives. Anger, for many of the children, represents such an emotion. The children need to be able to recognize anger in others, or even potential signs of anger, as their safety and wellbeing may depend on this ability. On the other hand, quick and efficient recognition of other emotions, including sadness, may be more difficult because they are not exposed to those emotions as often, and their adult caregivers are not adequately teaching them about those other emotions [24]. Whether similar difficulties emerge among children living in other highly compromised contexts is not clear. However, if parents are ill or have died and children are being raised without consistent adult input, they may not receive sufficient cues about emotions that would promote their recognition. This, in turn, may reduce their tendency to respond with empathy (see [25]).

Although such a possibility has yet to be tested directly, hints at its occurrence come from studies of empathy and prosocial behavior in high-risk youth that find young maltreated children are less likely than comparison children to help a peer in distress and more likely to react aggressively [26–28]. Also, children formerly exposed to war-related violence report less empathic responding and helping [29] than demographically similar children with no such exposure. A direct test of the links among adversity, empathic concern, and emotion recognition is needed.

We conducted such a test in the present study by assessing both high-risk and low-risk children’s and adolescents’ recognition of both clear and ambiguous emotional expressions and feelings of concern for the individuals displaying those expressions. We predicted that, compared to youth without a history of adversity exposure, youth exposed to chronic adversity would show reduced emotion recognition, with the exception of anger, relative to youth with no such exposure, and would report lower levels of empathic concern. We also expected that low emotion recognition would mediate the relationship between having a history of adversity exposure and empathic concern.

## Method

### Participants

One hundred twenty-three Swazi children and adolescents (“youth”), grades 5–12, ages 11–22, *M* = 14.04, 61 girls, served as participants. A majority of the sample was not in contact with their parent, due to the youth’s removal from home or parental death. The other youth were in school, often not in close proximity to their parents (e.g., some walked long distances to school, some lived with relatives or neighbors, and some lived with siblings). Thus, it was not feasible to obtain parental consent for the youth to participate. Instead, per our Institutional Review Board, formal approval to approach the youth was first granted by a professional responsible for the well-being of the youth in each area. This included the headmasters at the schools where youth were tested, the regional chief who oversaw education and well-being of individuals, including children, in his region, or the head social worker at the two foster care locations. In addition, on the days when data were collected, for ethical reasons, we also sought approval to approach youth from social workers or teachers who knew each youth personally. Once these individuals approved, we invited youth, who then provided written assent to participate. Two additional youth began the study but stopped part way through. Inclusion criteria were that the youth were in primary or secondary school and had no obvious cognitive disability. Social workers and teachers screened out youth with severe mental health problems.

Children were recruited from three types of environments. Two were characterized by high adversity. First, 47 “foster” youth were recruited from two out-of-home placement locations. These youth were included in light of extant literature showing biases in emotion recognition tendencies among children exposed to maltreatment or social deprivation [18, 21]. One set of foster youth (*n* = 33) came from a small rural town that has been converted to a large, live-in orphan village. Several hundred foster children live in small two-room cottages with five same age and gender peers and one unrelated live-in adult female caregiver. Youth had been removed from home or elected to leave as a result of exposure to maltreatment, sexual assault, or lack of adults in the homestead and were invited to live in the village (the process by which children were selected to come to the village is not known). The town is supported by private funds, but the staff work closely with governmental agencies to identify youth in need of placement and screen for appropriateness. Siblings may move to the village together but are rarely placed in the same cottage. The other out-of-home placement location (*n* = 14) was comprised of two residential facilities (one for boys and one for girls) in the capital of Swaziland. The facilities contain up to 14 same sex youth with at least one live-in female caregiver. A social worker also lives on site, and the locations regularly have staff from international charitable organizations visiting. In both locations, although the youth had previously been exposed to high levels of adversity, such as maltreatment or parental death, the youth were now in residential facilities that had running water inside, and all youth reported having a mattress on which they could sleep.

Second, 34 youth were recruited from one of two impoverished rural villages. These “rural” youth were attending the local primary school (grades 1–7). School was not in session, but 7^th^ graders were attending a class to prepare for their exit examination, and other youth were playing nearby at the request of the headmaster, who told them that we would be providing lunch. The youth in this group in many ways are similar to the youth in the foster group in that they were growing up in the same or similar regions. However, rural youth still lived in their home communities and their exposure to adversity was ongoing. For example, only 26% of these youth reported having running water in their homes, 23% reported not having a mattress or bed to sleep on, and 32% reported not getting enough food to eat on a regular basis. Given that their current state of poverty and adversity was likely much higher than the foster youth (even though prior exposure may have been similar), it was important to distinguish this group from the foster youth in the analyses.

Third, a sample of “comparison” youth (*n* = 42) was recruited from a well-funded private primary school in the capital. The comparison youth came from a variety of locations (some rural, some urban), but all were living with at least one biological parent. Moreover, their families were sufficiently well-off to pay the costs associated with private schooling and provide transportation for their youth to attend school. All but one of these youth had indoor running water, all had a mattress on which to sleep, and most (88%) reported getting enough food to eat. Thus, even though as a group, these youth may have been exposed to higher levels of challenge than youth in Western countries, the group was nonetheless considered middle class and included as an important lower-risk comparison group.

Data were collected over a two and a half-week period, with approximately three days spent per location, during which time we recruited and interviewed as many youth as possible. Far more youth wanted to participate than we were able to interview (e.g., upwards of 20–30 youth would be waiting to see if we had time to talk to them). We alternated selecting male and female youth to be interviewed, attempting to vary the ages while doing so. We provided snacks to all youth who were interested in the project, whether they were interviewed or not. The response rate of the youth who were invited was approximately 99%, and we completed 125 interviews. This sample size was sufficiently large to allow us to detect small to medium within-subject effects with power of .80. We also employed bootstrapping methods, a commonly applied strategy for enhancing power in mediation models, to help guard against potential violations of the assumption of multivariate normality in the analyses and generate a more accurate estimate of standard errors and confidence intervals for indirect effects [30]. Demographic details across the groups are presented in Table 1.

**Table 1:** Demographic characteristics.

Variables	Comparison (*n* = 42)	Foster *(n* = 47)	Rural Poor (*n* = 34)
Age in years	12.74^a^	14.85^b^	14.59^b^
Grade in school	7.12^a^	6.28^b^	6.79
Sex (% girls)	52.00	45.00	53.00
% with one or both parents deceased	7.32^a^	35.56^b^	29.41
Mean Adversity Index (age covaried)	0.16^a^	0.37^b^	0.38^b^

*Note*. Values with differing superscripts within rows are significantly different at *p* < .05 with the Bonferroni correction for multiple comparisons. The adversity score corresponded to the proportion of negative experiences in the family and community that the child reported had occurred out of all of those about which they were asked.

### Materials and procedure

Procedures were approved by the University Institutional Review Board, including procedures specific to approaching and interviewing youth in international settings. Testing was done in English, one of two official languages in Swaziland. The other official language is Swazi, and local interpreters (unknown to the youth) were available to elaborate in Swazi on some questions, as needed. Measures were administered via paper (*n* = 58, 47%) or tablet by one of three researchers. Interviews were audiotaped. Measures relevant to the current research are described here.

#### Demographic information, home, and community

Demographic questions asked about the youth’s age, year and month of birth, and grade in school. Adversity questions were included to confirm that the groups differed in levels of current adversity exposure, particularly when comparing the rural youth to the comparison youth. By virtue of the foster youth’s removal from home due to maltreatment or parental death and the fact that these youth had no alternative living arrangements available, they were assumed to have a history of adversity. Questions asked about the number and ages of individuals in the home and their relationship to the youth, the length of time in the current home, number of rooms, whether running water was currently available inside of the home, how many times a month the youth ate meat, whether the youth had a blanket or bed, and how the youth got to school (items adapted from The World Bank Child Needs Assessment Toolkit; [31]). Finally, yes/no questions asked about the community: whether robberies, assaults, domestic violence, alcohol and drug use, teen pregnancy, and violence against women had occurred (items adapted from the World Bank Social Capital Assessment Tool-Community Questionnaire; [32]).

#### Emotion recognition and empathic concern

A measure of emotion recognition and empathic concern was developed for the present study based on procedures in former studies concerning emotion understanding and empathy (e.g., [33–35]). Youth were shown images of scenes containing between one and five individuals (race matched that of our participants). The first and last images showed positive scenes (e.g., a family smiling). The other images showed negative scenes (e.g., a sick child with an intravenous drip, an adult crying) or ambiguous scenes (e.g., an adult pushing a cart of personal items looking in the distance). We classified images as positive, negative, or ambiguous based on whether a discrete emotional expression [36] was clearly depicted. If so, the images were classified as positive (happy) or negative (sad, fear, anger). The ambiguous images did not show the main character displaying a single or discrete emotional expression or showed a character displaying an expression inconsistent with the context.

Confirmation of the images’ and questions’ appropriateness and classifications came from several sources. An initial set of 26 images, all depicting individuals of African descent was shown to community leaders, teachers, and social workers in Swaziland for their feedback. Images deemed potentially confusing in terms of the content were eliminated. Question language was reviewed with these individuals as well, and phrasing was modified according to their suggestions. In prior work on empathic concern with children, approximately 20 images have been shown [37]. However, in this work, only one or two questions were asked about each image. Because we asked six questions per image, one of which required a narrative response, we elected to include a smaller number of total images, *n* = 11. Within these, as well, we retained a higher number of ambiguous (*n* = 6) images relative to positive (*n* = 2) and negative (*n* = 3), given our particular interest in variability in perceptions of ambiguity.

We also evaluated the comparison group’s ratings of the emotions depicted in the images as a second check on the images’ classifications. Given that this group had experienced the lowest levels of adversity, their responses could be considered a type of baseline or normative perceptions. The comparison group routinely rated characters in the negative images as high on one or more of the basic negative emotions and low on the positive emotion, and likewise, the positive images as only high on positive emotion and low on all negative emotions. Finally, this group’s mean ratings on the ambiguous images varied, but none was especially high or low. A third form of validation of the images came from ratings provided by an ethnically-diverse group of college students in the United States (*N* = 10, ages 20–26 years, 50% female). The students’ mean ratings of the characters in the images converged with those of the comparison youth: characters in the positive and negative images were rated as almost exclusively positive or negative, whereas characters in the ambiguous images were rated in the middle across emotions. In combination, these three checks on the images, in addition to evidence suggesting universality in emotion recognition abilities [38], suggested that they were appropriately tapping the desired emotions and were understandable to Swazi youth in the study.

Each image was presented individually and was followed by six questions. The first, designed to ensure that youth attended to each image, asked youth to describe what was happening in the image. After youth answered, the face and neck of the main character in the image was framed so that it was clearly distinguished from other information in the image. Youth were asked to look at the identified character and answer four questions, each on a 3-point scale (not at all, a little, a lot) how angry, happy, scared, and sad they thought that character in the image was (e.g., “How sad does this person feel?”) [33]. For the final question per image, youth were shown a 20-point pictorial scale (taken from [35]) with a cartoon face showing a large smile (score of 0) on one side and a large frown (score of 20) on the other (a neutral expression at 10 was also shown). Youth were told, “Sometimes when we see others, we feel good for them, sometimes we feel bad for them, and sometimes we don’t feel anything or we feel good and bad. Using this scale, point to the place that shows how you feel for the person in this box” (adapted from [35]). After the youth chose, the next image was presented. All questions, along with the pictorial rating scale, are provided in supplemental information.

Youth then completed other measures. At the end, they were thanked and children in the rural and foster groups were given snacks.

### Coding

Several composite scores were calculated. First, in order to confirm whether groups differed reliably in adversity exposure, the number of adverse experiences to which youth had been exposed in the home (e.g., not having running water in the home, one or both parents deceased) and community (e.g., drug use, violence against women) was summed and divided by the number possible to create an adversity index (*M* = .30, *SD* = .17).

Second, participants’ ratings on the 3-point scale of how happy, sad, angry, and afraid the character felt in each image were averaged within the three types of images: positive (*n* = 2), ambiguous (*n* = 6), and negative (*n* = 3). Finally, participants’ ratings of their empathic concern, that is, how good or bad they felt for the character (on the 20-point scale), were averaged separately for the three types of images (Positive: *M* = 6.70, *SD* = 5.00; Ambiguous: *M* = 13.76, *SD* = 2.85; Negative: *M* = 16.26, *SD* = 2.85).

## Analysis plan

Preliminary analyses included *t*-tests to assess whether mode of survey administration (tablet v. hard copy) influenced any of the participants’ responses, and analyses of variance to determine whether any group (comparison, foster, rural) differences emerged in demographic characteristics or life experiences. Next, mixed model analyses of covariance were conducted to examine whether the two adversity-exposed groups of youth differed in their perceptions of the emotions depicted by the main characters in the images. Group was entered as a between subject factor and the four emotion ratings were entered as the within subject dependent factor. Age was covaried. Separate models were conducted for the positive, ambiguous, and negative images. Significant effects with Huynh-Feldt corrections are reported, along with follow-up simple effects and pairwise comparisons using the Bonferroni adjustment, when appropriate. One-way analyses of covariance (age covaried) were conducted to assess whether the groups differed in their empathic concern for the characters in the positive, ambiguous, and negative images. Finally to test our main hypotheses, we conducted multiple mediation analyses using ordinary least squares path analysis with Hayes’ PROCESS macro for SPSS [39]. Groups were dummy coded, with the rural and foster groups being separately compared to the comparison youth. The ambiguous and negative images were examined in separate models. Bias-corrected bootstrap confidence intervals for the indirect effects were obtained. In each model, 10,000 bootstrap resamples were collected to estimate confidence intervals. All significant effects are reported. The *n*s vary slightly across analyses because a few youth skipped some questions.

## Results

### Preliminary analyses

We first compared youth’s mean ratings of character’s emotion and empathic concern based on whether youth completed measures via hardcopy or tablet. For the ambiguous images, participants rated the characters as slightly more happy, but also angry when the images were presented on the tablet; and for the negative images, participants rated the characters as more angry, *t*s (113) ≥ 2.05, *ps* ≤ .045, *ds* ≥.38. No differences emerged for participants’ ratings of empathic concern. Although the reason for these differences is not clear, we nonetheless confirmed that all subsequent significant effects remained when measure format was taken into account. We return briefly to the issue of format in the Discussion.

When group comparisons were conducted on demographic and experiential features, no differences in gender emerged. The comparison youth were younger on average than the other groups but were also in a higher grade academically than the foster youth, *Fs* (2,120)≥ 3.34, *ps* ≤ .04, $\eta_{p}^{2}\geq \;.05$ (see Table 1). Also, the comparison group reported fewer total negative life experiences, at home and in their community, than the foster and rural groups did, *F* (2, 119) = 29.46, *p* < .001, $\eta_{p}^{2}=\;.33$, as would be expected. The rural and foster youth did not differ in the number of reported adverse experiences.

### Emotion recognition

**Fig 1:**
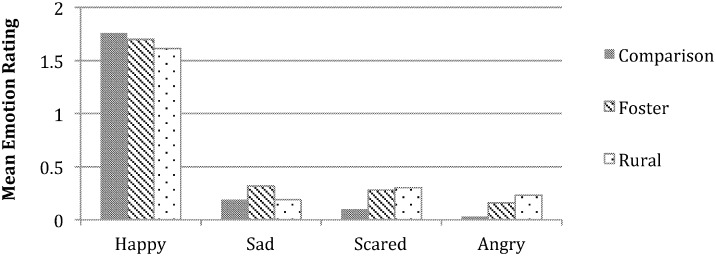
Group differences in emotion understanding for the positive images. Asterisks denote significant group differences within each emotion between the two adversity-exposed groups and the comparison group, post hoc *p*s < .05. Rating scales ranged from 0–2.

**Fig 2:**
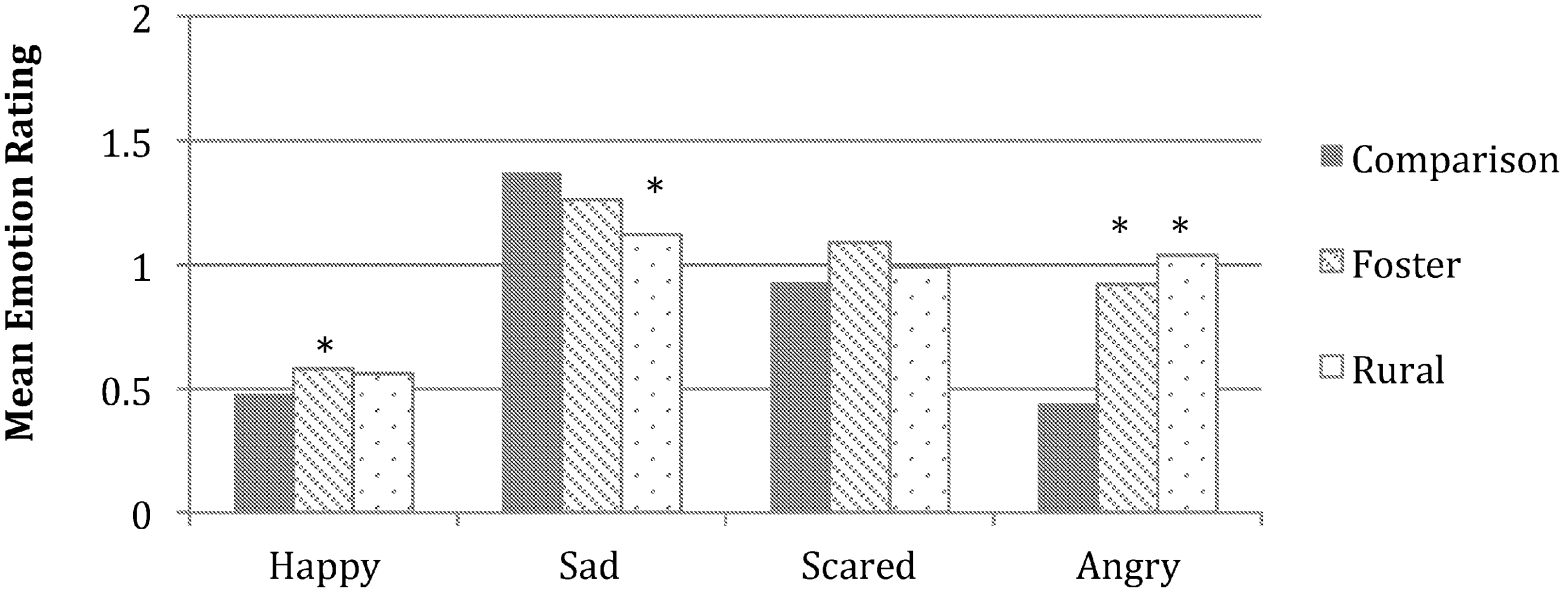
Group differences in emotion understanding for the ambiguous images. Asterisks denote significant group differences within each emotion between the two adversity-exposed groups and the comparison group, post hoc *p*s < .05. Rating scales ranged from 0–2.

**Fig 3:**
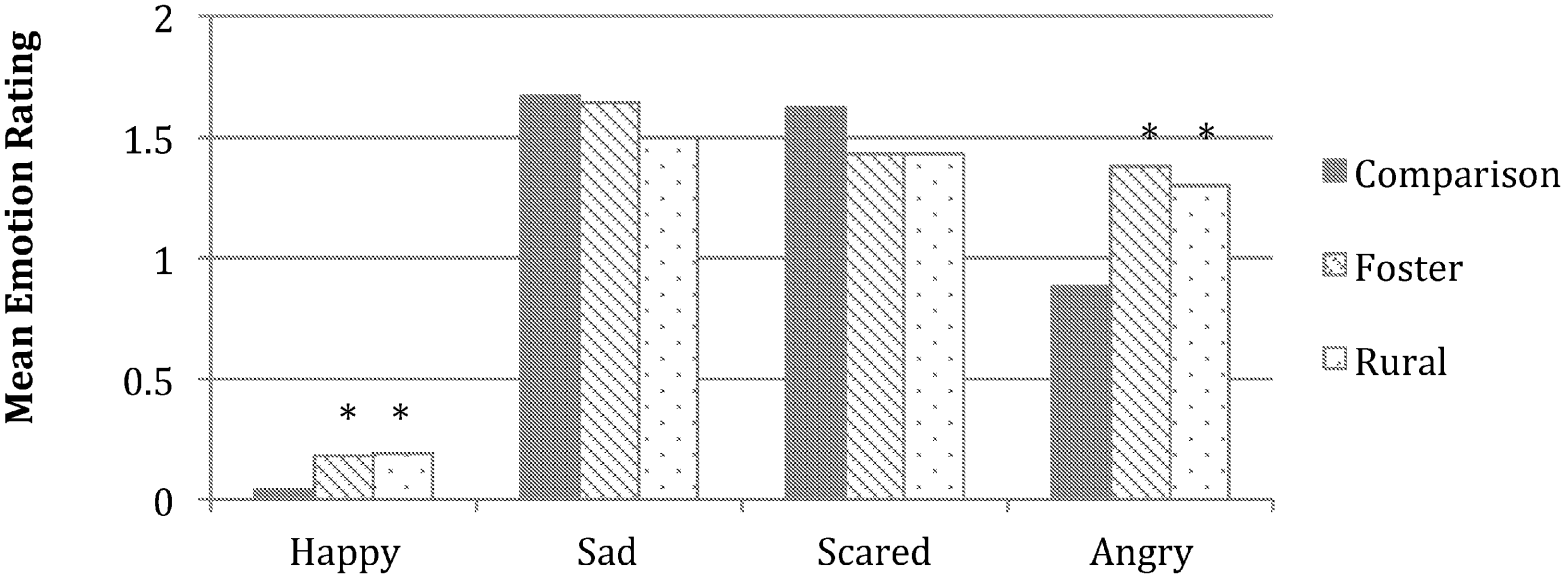
Group differences in emotion understanding for the negative images. Asterisks denote significant group differences within each emotion between the two adversity-exposed groups and the comparison group, post hoc *p*s < .05. Rating scales ranged from 0–2.

Youth ratings of the emotional displays depicted by the main characters in the images, separately for the foster, rural, and comparison samples, are presented in Figs 1–3. Mixed model ANCOVAs, conducted separately for the three types of images, revealed several significant effects. For positive images, the main effect of emotion was significant, *F* (2.18, 241.68) = 10.94, *p* < .001, $\eta_{p}^{2}=\;.09$. As seen in Fig 1, youth rated the main characters as substantially more happy than sad, angry, and fearful, with the latter three mean scores all falling near floor. No other significant effects emerged.

The ambiguous images were of particular interest, given the potential for high variability in youth’s interpretations [40]. When their ratings of the characters’ displays of the four emotions were entered into the ANCOVA, two interactions, emotion X age, *F* (2.60, 288.48) = 3.56, *p* = .02, $\eta_{p}^{2}=\;.03,$ and emotion X group, *F* (5.20, 288.48) = 7.10, *p* < .001, $\eta_{p}^{2}=\;.11,$, were significant. To analyze the emotion X age interaction, correlations were computed between participants’ emotion ratings and age. As age increased, participants’ ratings of the main character’s level of happiness decreased, *r* (115) = -.20, *p* = .04, and level of anger increased *r* (115) = .32, *p* = .001. To examine the emotion X group interaction, simple effects analyses were conducted by comparing the groups separately for each emotion. Groups differed in their ratings of the character’s happiness, sadness, and anger, *Fs* (2, 111) ≥ 3.18, *p*s ≤ .046, $\eta_{p}^{2}\geq \;.05$ (Fig 2). In partial support of our hypotheses, both foster and rural youth perceived greater anger in the characters than did comparison youth, *p*s < .001; and the rural youth perceived less sadness than the comparison youth, *p* < .05. In addition, although all youth’s ratings of the character’s happiness were low, the foster youth’s ratings of the characters in the the ambiguous images were somewhat higher than those of the comparison youth, *p* < .05.

When negative images were considered, the main effect of emotion, *F* (2.86, 317.50) = 4.50, *p* = .005, $\eta_{p}^{2}=\;.04,$ and emotion X group interaction, *F* (5.72, 317.50) = 5.76, *p* < .001, $\eta_{p}^{2}=\;.09,$ were significant. Follow-up analyses revealed that all groups reported very low levels of happiness in the main characters, but the adversity groups’ ratings were not quite as low as the comparison group’s ratings, *F* (2, 111) = 3.74, *p* = .03, $\eta_{p}^{2}s=\;.06$. Foster and rural groups also rated the main characters as more angry than the comparison group, *F* (2,111) = 7.13, *p* = .001, $\eta_{p}^{2}=\;.11$. Thus, to some extent, the adversity-exposed groups tended toward an anger attribution bias.

Second, perceptions of empathic concern, that is how good or bad youth felt for the characters in the positive, ambiguous, and negative images, were examined. Youth’s mean ratings of empathic concern, separated by image valence, were entered into separate 3 (group) ANCOVAs, age covaried. Group differences emerged for the ambiguous images, *F* (2, 111) = 2.95, *p* = .047. Follow-up comparisons revealed that rural youth, *M* = 12.75, reported feeling less bad for the main characters than comparison youth, *M* = 14.46, *p* < .05. The foster youth’s mean fell in between, *M* = 13.92, and did not significantly differ from the other group means.

### Adversity, emotion recognition, and empathy

Next, we tested empirically whether differences in youth’s emotion understanding, particularly differences across groups, contributed to group variations in empathic tendencies. The aforementioned analyses suggested that the groups differed primarily in perceptions of sadness and anger, and primarily with the ambiguous images, though to some extent also with the negative images. Because of these trends, only ratings of sadness and anger were included in subsequent analyses. The analyses consisted of multiple mediation analyses using ordinary least squares path analysis, Hayes’ PROCESS macro for SPSS [39]. Groups were dummy coded. The two high-adversity groups (rural and foster) were separately compared to the comparison youth. The ambiguous and negative images were examined in separate models. Bias-corrected bootstrap confidence intervals for the indirect effects were obtained. In each model, 10,000 bootstrap resamples were collected to estimate confidence intervals.

**Fig 4:**
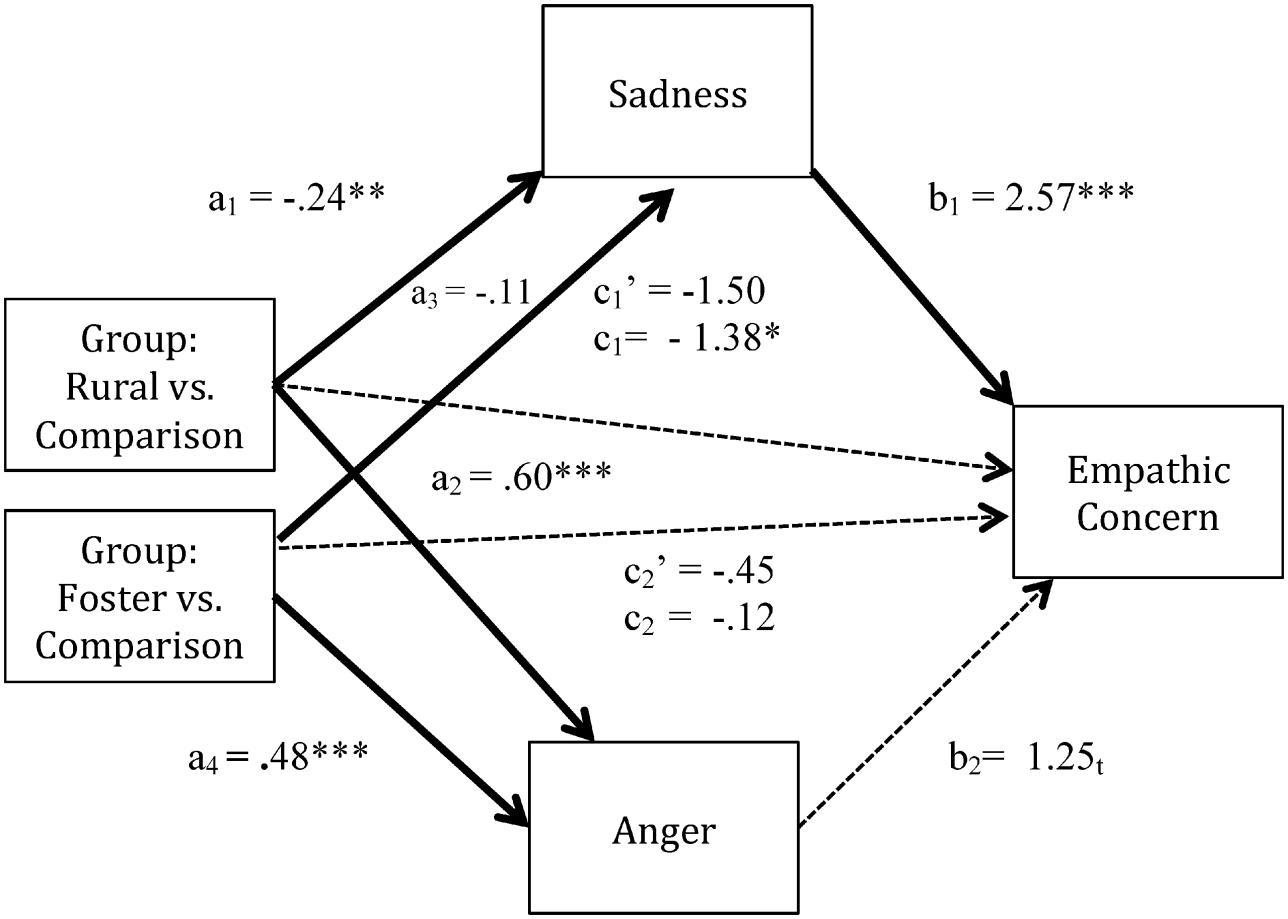
Multiple mediation model for the ambiguous images. Values shown are unstandardized regression coefficients, with significant paths, *p* < .05, bolded. * *p* < .05, ** *p* < .01, *** *p* < .001, *t* = .053 - .058.

First, analyses concerned youth’s perceptions of characters’ feelings of sadness and anger in the ambiguous images and youth’s ratings of empathic concern for those characters. Results confirmed hypotheses when comparing rural and comparison youth (Fig 4, Table 2). Relative to comparison youth, rural youth perceived less sadness in the ambiguous image characters, and in turn, youth who perceived less sadness reported feeling less bad for the characters. Stated another way, once the indirect path was taken into account, the direct path revealed that group differences in empathic concern were no longer significant. Rural youth also perceived more anger, but anger was not significantly related to how youth felt toward the character. Thus, perceived sadness was a significant mediator of the relation between adversity exposure (i.e., the rural group), and empathic concern with other potential predictors controlled. For foster compared to comparison youth, no evidence of mediation of perceived sadness or anger emerged.

**Table 2:** Mediational models of adversity exposure, sadness and anger, and empathic concern.

		Mediator 1 (Sadness) Estimate (SE)		Mediator 2 (Anger) Estimate (SE)		Total Effect Estimate (SE)		Direct Effect Estimate (SE)		Indirect Effect Estimate (95% CI)
a. Ambiguous Images										
Constant		1.37 (.06)***		.44 (.06)***		14.21 (.45)***		10.14 (.94)***		
Poor vs. Comparison	a_1_	-.24 (.09)**	a_2_	.60 (.10)***	c_1_	- 1.38 (.67)*	c_1_'	-1.50 (.77)	a_1_ ×b_1_	-.62 (-1.39, -0.17)*
									a_2_×b_2_	.75 (-0.05, 1.76)
Foster vs. Comparison	a_3_	-.11 (.09)	a_4_	.48 (.09)***	c_2_	-.12 (.62)	c_2_'	-.45 (.66)	a_3_×b_1_	-.28 (-0.81, 0.10)
									a_4_×b_2_	.60 (-0.02, 1.40)
Perceived Sadness							b_1_	2.57 (.67)***		
Perceived Anger							b_2_	1.25 (.65)		
b. Negative Images										
Constant		1.66(.07)***		.88(.09)***		16.03 (.46)***		12.58 (1.15)***		
Poor vs. Comparison	a_1_	-.20 (.09)*	a_2_	.42 (.14)**	c_1_	-.41 (.67)	c_1_'	.03 (.70)	a_1_×b_1_	-.42 (-1.04, -0.05) *
									a_2_×b_2_	-.02 (-0.40, 0.28)
Foster vs. Comparison	a_3_	-.02 (.09)	a_4_	.50 (.13)***	c_2_	.87 (.62)	c_2_'	.95 (.64)	a_3_×b_1_	-.05 (-0.50, 0.26)
									a_4_×b_2_	-.02 (-0.42, 0.36)
Perceived Sadness							b_1_	2.09(.65)**		
Perceived Anger							b_2_	-.05 (.46)		

*Note*. Unstandardized regression coefficients and corresponding standard errors are presented for the two multiple mediation models.

* p < .05

** p < .01

*** p < .001

**Fig 5:**
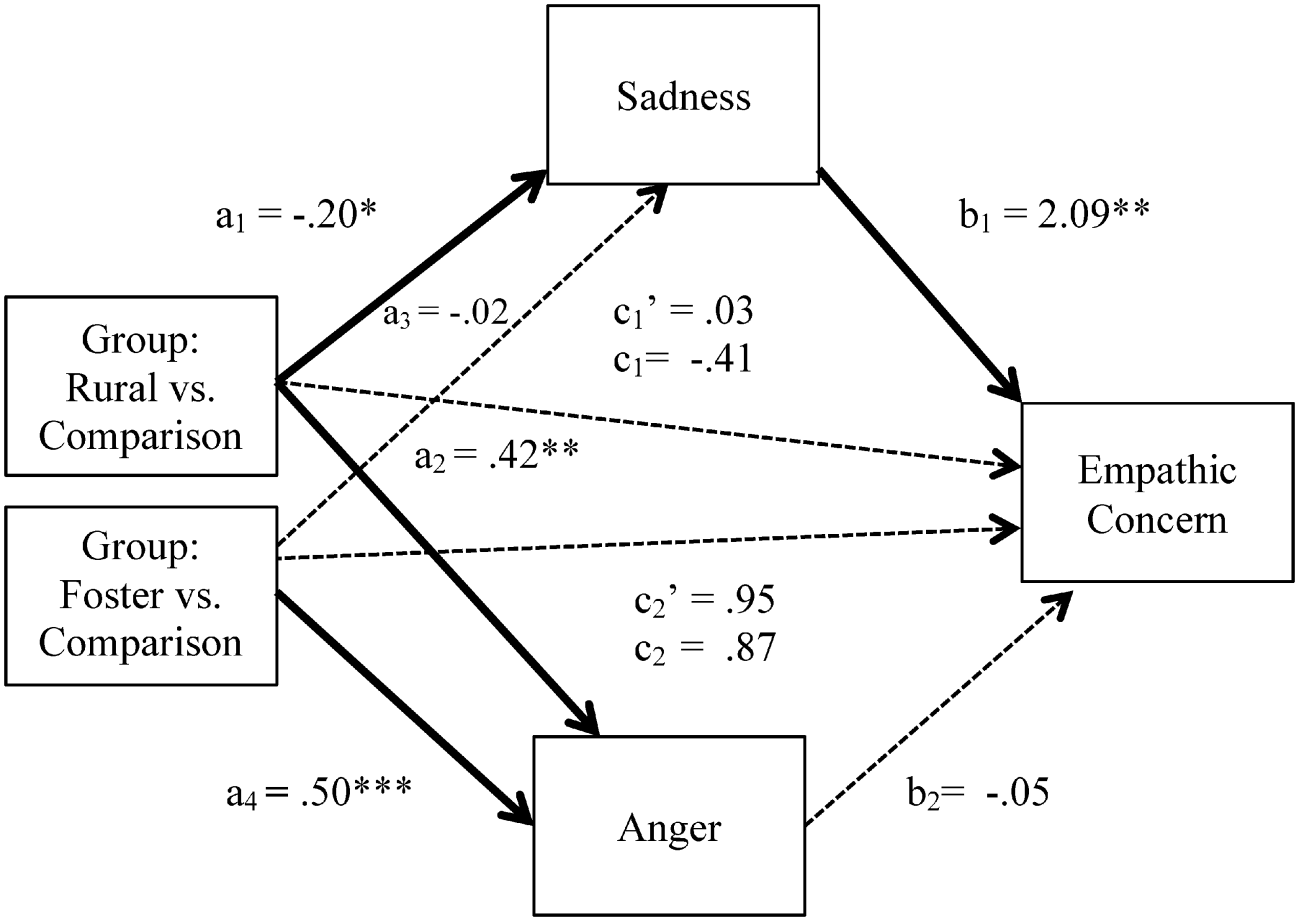
Multiple mediation model for the negative images. Values shown are unstandardized regression coefficients, with significant paths, *p* < .05, bolded. * *p* < .05, ** *p* < .01, *** *p* < .001.

Second, analyses were repeated for the negative images. Results were highly similar to those for ambiguous images; however, only for the rural youth (Table 2). Rural youth again perceived less sadness in the negative image characters than did comparison youth (Fig 5), and youth who perceived less sadness in the characters reported feeling less bad for them. The latter trend, as well, accounted for initial group differences in empathy. Rural youth also perceived greater anger than the comparison youth, but the association between perceived anger and empathic concern was non-significant. Models comparing foster and comparison youth were not significant.

In a final analysis, we re-evaluated our comparison group to determine whether their experiences of adversity mattered. That is, although youth in the comparison sample had experienced fewer negative life experiences and challenges than did the two other group (e.g., see Table 1), a sizeable minority of the comparison youth had lost a parent. We tested whether differences in emotion recognition, and the link between recognition and empathy, were due to the specific experience of parental loss. We divided the comparison and rural samples into two groups: youth who had versus had not lost one or both parents. We excluded the foster youth because they had the added experience of removal from home that may have differentially affected their reaction to the death of a parent. No evidence of mediation emerged for the negative and ambiguous images based on whether youth had versus had not experienced parental loss.

## Discussion

The overarching goal of the present study was to assess whether exposure to chronic adversity was associated with reduced empathic concern in youth, and test whether this association was mediated by variations in the youth’s capacity to recognize emotions in others. We pursued this goal by evaluating emotion recognition and empathic concern in a unique sample of Swazi youth, many of whom had experienced significant adversity in the past, and for some, at present. The results provide novel insight into how core emotional processes, namely emotion recognition and empathic concern, operate and potentially influence one another in high-risk youth growing up in environments characterized by uncertainty, loss, and possible deprivation.

Overall, our work suggests that youth who have experienced chronic hardship, especially when that hardship is ongoing, show different patterns of emotion recognition than youth who have not, and that these patterns may alter the level of empathic concern children express toward others. Specifically, adversity-exposed youth perceived more anger in images showing negative expressions, but also in ambiguous images in which the main character’s expression was not entirely clear. These data align with prior work showing heightened sensitivity to the perception of anger in youth growing up in harsh environments [18, 21]. This anger bias may well be adaptive in the youth’s compromised contexts where vigilance toward threat is imperative [41]. Over time, this vigilance may develop into a hostile attribution bias even when threat is no longer present [19, 42], the latter of which is believed to place children at risk for increased aggression, delinquency, poor peer relationships, and anxiety and depression [43, 44]. Biases in youth’s ability to recognize anger in others may therefore have critical implications for a host of behavioral and emotional problems, one of which includes a reduction in empathic concern.

Youth exposed to adversity also tended to perceive less sadness in the images depicting negative and ambiguous expressions. This reduced ability is consistent with findings of research concerning emotion processing tendencies in youth exposed to other types of adversity. Former child soldiers, young war and terrorism survivors, and maltreated children all exhibit similar deficits in understanding sadness in others [23, 45]. Moreover, in the present study, when empathic concern was considered, these differences in youth’s perceptions of characters’ sadness were key. Less recognition of sadness was associated with less reported empathic concern, particularly in youth who continue to live in chronically deprived settings. This suggests that chronically deprived youth may not be less empathic than more advantaged youth per se, but rather, may have difficulties recognizing emotions that provoke feelings of concern, which in turn reduces the need or opportunity to feel empathic. Such a possibility aligns with past theory and work that contends that perceiving distress in others, particularly sadness, is critical in motivating empathic responding, affiliation, and prosocial behavior [2, 46, 47].

Several other interesting trends in these data are also noteworthy. For one, there were slight differences among groups’ perceptions of happiness in the images displaying negative and ambiguous expressions. Foster and rural youth, that is, both groups with a history of chronic adversity, perceived greater happiness in some of these images than comparison youth, although as already noted, all youths’ ratings were fairly low. On the one hand, this finding is inconsistent with past research, which suggests that emotion biases in youth growing up in adverse contexts are specific to cues of anger or negative emotions, rather than happiness [48, 49]. On the other hand, these trends hint that adversity-exposed youth may be more confused about emotions generally and hence see even conflicting types of emotions (happiness and anger) in others. Finally, it is possible that the high-adversity youth were more likely than comparison youth to perceive positivity in negative or ambiguous expressions due to contextual or experiential differences. Nonetheless, future work should examine this pattern in greater detail to better understand the extent to which life experiences influence youth’s perception of happiness in others, as well as the implications of these perceptions for youth development.

Our data also suggested that youth’s perceptions of anger in others might influence empathic concern for both adversity-exposed groups, but not the comparison group. Foster and rural youth perceived greater anger in ambiguous expressions relative to comparison youth and this tendency was associated with reporting greater empathic concern for the characters in these images. It is unclear why perceiving greater anger, an often outwardly hostile emotion, would affect youth’s empathic concern in a positive direction. Perhaps, for adversity-exposed youth, anger served as a motivator, just as anger often acts as an approach emotion, leading to action. In this specific paradigm, that action was expressed as concern for the main character. It is also possible that the images may have evoked feelings of outrage in some adversity-exposed youth. Such feelings have been associated with prosocial and moral behaviors, at least under certain conditions [50]. Overall, these findings highlight that a similar outcome—in this case, empathic concern—might be motivated by different processes depending on children’s prior exposure to adversity. Alternatively, perhaps perceiving high levels of any negative emotion leads to increases in empathic feelings, though which negative emotions are perceived seem to vary depending on adversity exposure. Future research should consider these possibilities more systematically, given that the relations between anger perception and empathic concern only emerged at trend levels in the present data.

It is worth commenting on two other trends that emerged. First, intriguing differences emerged between the two adversity groups in their perceptions of emotions and the meditational role of emotion recognition. In addition, differences in perceptions emerged between children who completed the measure on a tablet or by looking at hard copy images. Regarding the adversity groups, the rural and foster groups differed in relation to some specific emotion recognition patterns and associations among adversity. The rural youth were presently living in impoverished conditions with limited access to running water and in some cases, adequate meals and shelter; moreover, many had lost parents and family members due to HIV/AIDS or other illness, and thus faced unstable or inconsistent caregiving. The foster youth, though originally from areas similar to the rural youth, had been selected for placement in the foster villages. Once there, the youth were cared for by live-in caregivers (who stayed with the youth for several years at a time) and social workers. The foster youth had access to schools, clothing, supplies, and reliable food and water. Although they had left their primary home and hence changed caregivers completely, the youth were now surrounded by a set of consistently available adults who provided emotional support and guidance. This level of all-encompassing intervention may well alter, perhaps in positive ways, youth’s feelings of empathy even if the intervention does not fully change emotion recognition tendencies. Indeed, there is evidence that interventions focused on relationship-building have benefits for youth’s emotional functioning and subsequent social relationships [51, 52]. How these and other changes in context affect youth’s empathy and concern for others is a crucial area for further inquiry.

Regarding the study methods, youth who saw images on the tablet rated the characters as slightly more angry and happy in the ambiguous images and slightly more angry in the negative images than youth who saw hard copy pictures. Children were able to change the size of the images with the tablet but not the hard copy, which could have affected their responses. However, it is not clear why changing the image size differentially affected only perceptions of anger and happiness and only of some images. Nonetheless, as technology becomes increasingly used in data collection around the globe, including with children and including in relation to their understanding of emotional displays in others, it will be important to consider how technology may influence responding.

Although the study’s findings are exciting and novel, the conclusions are tentative without further exploration of several key issues that could not be addressed with the current methodology. For one, it will be important to assess the extent to which cognitive ability and other developmental processes shape children’s emotion recognition tendencies and feelings of empathic concern. The comparison youth were, on average, younger than both adversity-exposed groups but at the same time in a higher grade academically than the foster youth. Even though studies examining emotion recognition in other groups of adversity-exposed youth (e.g., maltreated; [18, 22]) have controlled for cognitive ability and still found differences when comparing those youth to community samples, cognitive ability could still indirectly influence youth performance, for instance, by affecting their willingness to answer some questions in a comprehensive or detailed manner. In addition, it was not possible to standardize our measures. However, we made efforts to confirm that the images captured the emotions intended by showing the images to other populations and by examining the responses of youth in the comparison group. Moreover, there is some level of universality in humans’ ability to recognize emotional expressions in others, and these abilities are often strongest when viewing expressions presented by individuals from similar ethnic and racial backgrounds [38]. All of our images conformed to the latter and depicted African individuals displaying emotions. Nonetheless, future research should include a wider array of emotion recognition tasks, including those that have been standardized for specific races and ethnic groups. Future research should also assess whether similar findings emerge in different contexts and that include youth exposed to other forms of adversity (e.g., chronic health problems, war exposure) to determine the conditions under which variations in emotion recognition, particularly in ambiguous situations, relate to or influence empathic concern. Such research will reveal the extent to which the links between emotion recognition and empathic concern are generalizable versus context-specific.

In closing the current study offers novel insight into potential processes underlying empathy in high-risk youth and has implications for interventions aimed at increasing empathic responding in children and adolescents. What emotions youth perceive in others, which is directly related to their level of adversity, predicts the extent to which youth feel empathic concern for those others. More broadly, emotion recognition may serve as a key component to appraisal processes that, in turn, motivate empathic behaviors [53]. Insofar as it is possible to alter children’s interpretations of ambiguous expressions [54], and that these alterations may affect behaviors (e.g., aggression), it may be possible to begin to shape as well, empathic concern and perhaps even helping or prosocial behaviors. Overall, this line of work has tremendous potential to enhance understanding of the processes by which people support and engage with others versus disconnect, especially in situations of challenge, in which connection and cooperation may be vital to resilience and even survival.
